# The multifaceted responses of primary human astrocytes and brain microvascular endothelial cells to the Lyme disease spirochete, *Borrelia burgdorferi*

**DOI:** 10.1042/AN20130010

**Published:** 2013-08-16

**Authors:** Catherine A. Brissette, Eric D. Kees, Margaret M. Burke, Robert A. Gaultney, Angela M. Floden, John A. Watt

**Affiliations:** *Microbiology and Immunology, University of North Dakota School of Medicine and Health Sciences, Grand Forks, ND 58203, U.S.A.; †Department of Anatomy and Cell Biology, University of North Dakota School of Medicine and Health Sciences, Grand Forks, ND 58203, U.S.A.

**Keywords:** astrocyte, blood–brain barrier, *Borrelia burgdorferi*, chemokine, endothelium, neuroborreliosis., BBB, blood–brain barrier, CCL, CC chemokine ligand, CNS, central nervous system, CSF, cerebrospinal fluid, CXCR, CXC chemokine receptor, ECM, endothelial cell medium, GAPDH, glyceraldehyde-3-phosphate dehydrogenase, HBMEC, human brain microvascular endothelial cells, IL, interleukin, MOI, multiplicity of infection, QPCR, quantitative PCR, TNFα, tumor necrosis factor α

## Abstract

The vector-borne pathogen, *Borrelia burgdorferi*, causes a multi-system disorder including neurological complications. These neurological disorders, collectively termed neuroborreliosis, can occur in up to 15% of untreated patients. The neurological symptoms are probably a result of a glial-driven, host inflammatory response to the bacterium. However, the specific contributions of individual glial and other support cell types to the pathogenesis of neuroborreliosis are relatively unexplored. The goal of this project was to characterize specific astrocyte and endothelial cell responses to *B. burgdorferi*. Primary human astrocytes and primary HBMEC (human brain microvascular endothelial cells) were incubated with *B. burgdorferi* over a 72-h period and the transcriptional responses to the bacterium were analyzed by real-time PCR arrays. There was a robust increase in several surveyed chemokine and related genes, including IL (interleukin)-8, for both primary astrocytes and HBMEC. Array results were confirmed with individual sets of PCR primers. The production of specific chemokines by both astrocytes and HBMEC in response to *B. burgdorferi*, including IL-8, CXCL-1, and CXCL-10, were confirmed by ELISA. These results demonstrate that primary astrocytes and HBMEC respond to virulent *B. burgdorferi* by producing a number of chemokines. These data suggest that infiltrating phagocytic cells, particularly neutrophils, attracted by chemokines expressed at the BBB (blood–brain barrier) may be important contributors to the early inflammatory events associated with neuroborreliosis.

## INTRODUCTION

Many microorganisms can cause inflammation in the CNS (central nervous system) by damaging the protective barriers that sequester the brain from the periphery. These ‘neurovascular units’, which include vascular endothelia, glia, and neurons, work together to maintain normal brain function (Grab et al., 2011). Breakdown of the BBB (blood–brain barrier) and blood–CSF (cerebrospinal fluid) barrier can facilitate entry of both microorganisms and activated immune cells into the CNS. Of critical importance to the development and maintenance of the BBB is the astrocyte. Astrocytes are glial cells that vastly outnumber neurons in the brain. Astrocytes are involved in metabolic interactions with neurons, and also form close associations with endothelia and fibroblasts (Magistretti and Ransom, 2002). The interaction of astrocytic endfeet with the endothelium of the BBB is key to the induction of many barrier properties, including tight junction formation and the expression and localization of transporters. Astrocytes and the endothelium of the BBB respond to various insults by producing a wide range of chemokines and neurotrophic agents. Their close association with many cell types of the CNS and its barriers highlights the importance of understanding the response of astrocytes and brain endothelial cells to pathogens that cause neuroinflammation.

Over 90% of all arthropod-borne diseases in the USA is caused by the spirochete *Borrelia burgdorferi* (Radolf et al., 2012). *B. burgdorferi* is the agent of Lyme disease, which can manifest in skin, joints, the heart, and the brain. Neuroborreliosis, which occurs in up to 15% of untreated patients (Hildenbrand et al., 2009), results in meningitis, headache, and facial nerve palsy (Rupprecht et al., 2008). Peripheral nerve disease is also common, and long-term symptoms remain in up to 50% of patients even 5 years post-treatment (Ljostad and Henriksen, 2008). The ability of *B. burgdorferi* to infect immunocompetent humans and other vertebrates for extensive periods of time, (Moody et al., 1990; de Souza et al., 1993; Steere, 2001; Miller et al., 2003; Stanek and Strle, 2003) coupled with the potential for long-term sequelae, make this disease particularly insidious.

The Lyme disease spirochete has an affinity for the CNS (Rupprecht et al., 2008). *B. burgdorferi* can be isolated from the CSF of humans as early as 18 days after the bite of an infected tick (Fallon et al., 2010). In a rhesus macaque model, the spirochete can be detected in the leptomeninges, dorsal root ganglia, and occasionally the parenchyma of the brain (Cadavid et al., 2000). *B. burgdorferi* has the potential to cross the BBB and come into direct contact with the brain microvascular endothelium and astrocytes. The spirochete freely crosses brain vascular endothelial cells in the presence of the proenzyme plasminogen (Grab et al., 2005). Despite the potential for access and interaction of *B. burgdorferi* with glia, almost nothing is known about the interaction of *B. burgdorferi* with human astrocytes. In a single published study in which the Lyme spirochete was incubated with human astrocytes, the authors found a significant up-regulation of MMP-9 (matrix metalloprotease 9), which may play a role in the breakdown of brain barriers (Perides et al., 1999). Several studies with murine and non-human primate astrocytes, however, suggest that these cells could play important roles in the innate immune response to *B. burgdorferi.* For example, *B. burgdorferi* induces IL (interleukin)-6, IL-10, and TNFα (tumor necrosis factor α) from murine astrocytes (Chauhan et al. 2008; 2009), and up-regulates the pattern recognition receptor NOD2 (Sterka et al., 2006). Astrocytes from non-human primates respond to *B. burgdorferi* by up-regulating IL-6 and the chemokines IL-8, CCL (CC chemokine ligand) 3 and CCL4 (Bernardino et al., 2008).

Similarly, while the response of heart and umbilical endothelial cells to *B. burgdorferi* has been examined, little is known about *B. burgdorferi* interaction with HBMEC (human brain microvascular endothelial cells), and to our knowledge, no studies have focused on primary HBMEC (Boggemeyer et al., 1994; Sellati et al., 1995; Ebnet et al., 1996; Burns et al., 1997; Gebbia et al., 2001; 2005; Dame et al., 2007; Ramesh et al., 2009).

Because of the critical roles of astrocytes and HBMEC in sensing, responding, and adapting the neural environment to pathogens, and the paucity of data in this regard, we wanted to delineate the astrocytic response to *B. burgdorferi*. We were particularly interested in the expression of chemokines because of their clear import to the trafficking of leukocytes to the site of infection. In this work, we demonstrate that *B. burgdorferi* up-regulates the expression of several key chemokines specifically from human astrocytes and HBMEC.

## MATERIALS AND METHODS

### Primary cultures of human astrocytes

Primary cultures of human astrocytes were obtained from ScienCell Research Laboratories (catalog no.1800) and maintained on poly-l-lysine coated flasks (2 μg/cm^2^, T-75) in the astrocyte medium (ScienCell, catalog no. 1801). To stimulate the cells, astrocytes were used between passages 3 and 4 at 75% confluence. Prior to *Borrelia* stimulation, medium was replaced with antibiotic-free astrocyte medium. Astrocytes were stimulated with *B. burgdorferi* at an MOI (multiplicity of infection) of 40:1 for 6–72 h.

### Primary cultures of HBMEC

Primary HBMEC were obtained from ScienCell Research Laboratories (catalog no. 1000) and maintained on fibronectin coated flasks (2 μg/cm^2^, T-75) in ECM (endothelial cell medium, ScienCell; catalog # 1001). To stimulate the cells, HBMEC were used between passages 3 and 4 at 75% confluence. Prior to *Borrelia* stimulation, ECM was replaced with ECM minus antibiotics. HBMEC were stimulated with *B. burgdorferi* at a MOI of 40:1 for 6–72 h.

### Bacterial culture

Virulent *Borrelia burgdorferi* strain B31 MI-16 (Casjens et al. 2000; Fraser et al. 1997; Miller et al., 2003) was grown at 34°C to cell densities of approximately 1×10^7^ /ml in modified BSK-II (Barbour-Stoenner-Kelly II) medium (Zückert, 2007). *B. burgdorferi* was pelleted at 6000 ***g***, washed three times with PBS, and resuspended in DMEM (Dulbecco's modified Eagle's medium) without antibiotics. *B. burgdorferi* were enumerated by darkfield microscopy using a Petroff-Hausser chamber. For stimulation experiments, *B. burgdorferi* was used at a MOI of 40:1. Total DNA (chromosomal and plasmids) was isolated using the DNeasy blood and tissue kit (Qiagen) according to the manufacturer's instructions. Spirochete viability in the presence of cells and culture medium was monitored by total motile spirochetes by counting ten random fields via darkfield microscopy (Supplementary Figure S1 at http://www.asnneuro.org/an/005/an005e119add.htm).

### RNA isolation and cDNA synthesis

RNA was isolated using the RNeasy kit (Qiagen) according to the manufacturer's instructions. Briefly, after aspiration of media, flasks were washed three times with warm sterile PBS. Cells were detached using Trypsin–EDTA (ScienCell), trypsin activity was neutralized with the addition of ECM plus FBS, and the resulting cell slurry was homogenized by passing through gradually smaller sterile glass pipettes. Cells were lysed in Buffer RLT (Qiagen). Genomic DNA was removed by on-column DNA digestion with RNase-Free DNase Set (catalog no. 79254, Qiagen). Following isolation, RNA was concentrated using Qiagen RNeasy MinElute Cleanup Kit (catalog no. 74204). RNA quality and concentration was assessed spectrophotometrically on a Bio-Tek Epoch (Bio-Tek) and cDNA synthesis was performed using Qiagen RT^2^ First Strand Kit (Catalog no. 330401) according to the manufacturer's instructions.

### QPCR (quantitative PCR)

The expression of multiple genes was analyzed using RT^2^ Profiler PCR arrays (SABiosciences; Human Chemokines and Receptors, catalog no. PAHS-022) on a BioRad myIQ2 real-time PCR instrument. Data were analyzed using SABiosciences software. Changes in individual genes were confirmed using individual PCR primer sets (SABiosciences; Table 1). Briefly, each reaction contained 5.5 μl nuclease-free H_2_O, 2 ml primer mix at 10 μM, and 12.5 μl BioRad SyberGreen Supermix ±5 μl template DNA or water (no template control). The QPCR was performed in 40 cycles following an initial 10 min denaturation at 95°C. Each cycle consisted of a 1 min annealing step performed at 60°C, followed by a 15 s melting interval at 95°C. Product melting curves were generated at the end of the reaction using a stepped temperature gradient of 0.5°C×10 s starting at 60°C. Expression levels of all transcripts were compared with housekeeping genes (β-actin and GAPDH (glyceraldehyde-3-phosphate dehydrogenase)) and the relative changes in gene expression were compared with those of untreated cells using the 2^−ΔΔCT^ method where C_T_=threshold cycle. This method was used on each individual example with the untreated sample as the comparator (Schmittgen and Livak, 2008). All samples were analyzed in triplicate from at least two independent biological replicates per time point.

**Table 1:** Primer sets used for QPCR

Primer name	RefSeq accession no.	SABiosciences cat no.
β-actin	NM_001101	PPH00073G
GAPDH	NM_002046	PPH00150F
IL-8	NM_000584	PPH00568A
CXCL-1	NM_001511	PPH00696C
CXCL-10	NM_001565	PPH00765E

### ELISA

Culture supernatants were removed after stimulation and stored at −80°C. ELISA for IL-8, CXCL-1, and CXCL-10 was performed according to the manufacturer's instructions (R&D Systems). Briefly, all reagents were brought to room temperature and prepared as instructed. Plates were coated overnight with 100 μl of appropriate capture antibody. Following aspiration and wash, 100 μl of appropriate chemokine standards, controls, or sample were added to each well. Plates were incubated for 2 h at room temperature. Following aspiration and wash, 100 μl of antibody conjugate was added to each well, followed by 2 h incubation at room temperature. Following aspiration and washes, the chemokine of interest was detected by adding a chromogenic substrate followed by a stop solution. Plates were read at an optical density of 450 nm on a BioTek Epoch plate reader. Samples were run in triplicate and data pooled from each treatment group. Data represent the means±S.E.M. from two independent biological replicates analyzed in triplicate per time point.

### Statistical analysis

For ELISA and QPCR, each experiment of cell stimulation with bacteria was carried out two times in independent experiments with triplicate replicants. Results are presented as means±S.E.M. and were analyzed by Student's *t* test. Differences in values are considered significant at *P*<0.05. *P*<0.05=; *P*<0.01=**; *P*<0.0005=***; *P*<0.0001=****. For QPCR, the values are normalized to the negative control (medium alone) and shown as the fold number of the control's value.

## RESULTS

### Transcriptional profiling of chemokine and receptor gene expression in human astrocytes and HBMEC in response to *B. burgdorferi*

To begin to delineate the responses of human astrocytes to *B. burgdorferi*, we stimulated primary cultures of normal human astrocytes or HBMEC with exponential phase spirochetes for 6, 12, 24 or 72 h. At least two independent stimulation experiments were performed per cell type, with three replicates per experiment. After RNA extraction from stimulated and control cells, cDNA was synthesized and used as a template for commercial real-time PCR array analysis. Commercial QPCR arrays were used to quantify the transcription of a panel of human chemokines, chemokine-like genes, and their receptors. Threshold cycle values across experiments and between replicates were very reproducible, with standard deviations averaging about 1 cycle across the array (results not shown). Data were analyzed and fold changes between control and treated samples using the manufacturer's software (SABiosciences), with a threshold of 3-fold change in gene expression. For both astrocytes and HBMEC, most of the transcriptional changes occurred early, at 6 h post-stimulation. In the case of astrocytes, out of 84 genes represented on the array, only eight genes (10.5%) were differentially regulated with a fold change above 3 at 6 h. Genes up-regulated more than 3-fold in all biological replicates at 6, 12, 24 or 72 h are shown in Table 2. Interestingly, very few genes were down-regulated in response to *B. burgdorferi* stimulation. At 12 h, CCL11 (eosinophil chemoattractant and CCL4 (NK (natural killer) cell and monocyte chemoattractant) were down-regulated 6- and 3.2-fold, respectively. At 24 h post-stimulation, the chemokine receptor CXCR (CXC chemokine receptor)3 was down-regulated 4.3-fold, and at 72 h, the chemokine receptor CXCR4 was down-regulated 3-fold while the neutrophil chemoattractant IL-8 was down-regulated 4.9-fold, in relation to unstimulated cultures. Similarly, for primary endothelial cells, only a small fraction of genes were differentially regulated at any time point examined (Table 3).

**Table 2:** Transcriptional profiling of chemokine and cytokine gene expression in astrocytes in response to *B. burgdorferi* Astrocytic responses to *B. burgdorferi*. At least two biological replicates were performed per time point. Values shown correspond to the mean ratio of triplicate measurements determined between normalized gene intensity values after 6–72 h of stimulation with 40:1 *B. burgdorferi* strain B31 compared with gene intensity values from unstimulated cells. Only genes which demonstrated at least a 3-fold change in all biological replicates are included.

(a) 6 h post-stimulation		
Gene	Fold regulation	Description/function
CXCL-1	9.2	Neutrophil chemoattractant
CXCL-2	4.3	Chemokine
CXCL-3	5.5	Monocyte chemoattractant
CXCL-6	3.8	Chemokine, T-cell chemotaxis
CXCL-10	3.6	Monocyte, NK, T-cell migration
CXCL-12	3.7	Lymphocyte chemoattractant
IL-8	9.1	Neutrophil chemoattractant
TNFα	8.8	Cytokine
(b) 12 h post-stimulation		
Gene	Fold regulation	Description/function
CXCL-1	3.4	Neutrophil chemoattractant
CXCL-10	3.5	Monocyte, NK, T-cell migration
CXCL-12	3.1	Lymphocyte chemoattractant
IL-8	4.3	Neutrophil chemoattractant
(c) 24 h post-stimulation		
Gene	Fold regulation	Description/function
CXCL-1	7.9	Neutrophil chemoattractant
CXCL-2	8.1	Chemokine
CXCL-3	9.9	Chemokine
CXCL-6	11.9	Chemokine, T-cell chemotaxis
CXCL-10	26.7	Neutrophil chemoattractant
IL-8	13.6	Neutrophil chemoattractant
(d) 72 h post-stimulation		
Gene	Fold regulation	Description/function
CCL8	3.1	Monocyte chemoattractant
CXCL-12	5.1	Chemokine
TLR2	4.1	Pattern recognition receptor

**Table 3:** Transcriptional profiling of chemokine and cytokine gene expression in HBMEC in response to *B. burgdorferi* HBMEC responses to *B. burgdorferi*. At least two biological replicates were performed per time point. Values shown correspond to the mean ratio of triplicate measurements determined between normalized gene intensity values after 6-72 h of stimulation with 40:1 *B. burgdorferi* strain B31 compared with gene intensity values from unstimulated cells. Only genes that demonstrated at least a 3-fold change in all biological replicates are included.

(a) 6 h post-stimulation		
Gene symbol	Fold regulation	Description/function
CXCL-1	8.6	Chemokine, neutrophil chemoattractant
CXCL-2	14.7	Chemokine
CXCL-3	11.4	Chemokine
IL-8	3.2	Chemokine, neutrophil chemoattractant
TNFα	4.0	Cytokine
(b) 12 h post-stimulation		
Gene symbol	Fold regulation	Description/function
CXCL-2	24.9	Chemokine
CXCL-6	11.3	Chemokine
IL-8	22.6	Chemokine, neutrophil chemoattractant
(c) 72 h post-stimulation		
Gene symbol	Fold regulation	Description/function
CXCL-6	95.8	Chemokine; T-cell chemotaxis
CXCL-10	327.0	Chemokine; monocyte, NK, T-cell migration

### Validation of selected genes among those found to be differentially regulated by QPCR array analysis

The mRNA expression of selected genes (IL-8, CXCL-1, and CXCL-10) was analyzed in kinetic experiments at 6, 12, 24, and 72 h after *Borrelia* stimulation. Expression levels of all transcripts were compared with housekeeping genes (β-actin or GAPDH) and the relative changes in gene expression were compared with those of untreated cells using the 2^−ΔΔCT^ method on each individual example with the untreated sample as the comparator. Results obtained after β-actin and GAPDH normalization were very similar to each other (results not shown). A similar trend in transcriptional induction was observed by QPCR and that seen in the commercial real-time PCR arrays. For astrocytes, we confirmed strong up-regulation of the genes encoding IL-8 and CXCL-1, with highest transcription detected at 12 h, and CXCL-10, with highest transcription detected at 24 h post-stimulation. For HBMEC, transcription of IL-8, CXCL-1, and CXCL-10 all peaked at 72 h.

### Protein expression in *B. burgdorferi* stimulated astrocytes and HBMEC

To confirm whether *B. burgdorferi* induce chemokine production and secretion after co-incubation with primary human astrocytes, we measured IL-8, CXCL-1, and CXCL-10 synthesis. For astrocytes, chemokines IL-8 and CXCL-1 were secreted in time-dependent manner, with peak secretion at 24 h after cell stimulation compared with unstimulated control cells. CXCL-10 expression, in contrast, was elevated as early as 6 h post-stimulation and remained relatively unchanged at subsequent time points examined (Figure 2). Interestingly, although *B. burgdorferi* viability in the presence of astrocytes and cell culture medium decreased over time, heat-killed bacteria were still able to induce a significant chemokine response at 24 h (Supplementary Figure S1).

**Figure 1:**
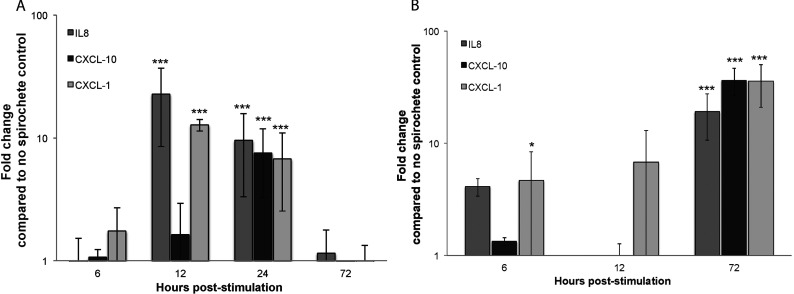
Validation of selected genes among those found to be differentially regulated by QPCR array analysis (**A**) Primary human astrocytes. (**B**) Primary HBMEC; all cells were stimulated with *B. burgdorferi* for 6–72 h. RNA was extracted and cDNA synthesized. Individual primer sets (SABiosciences) were used to amplify transcripts of interest by QPCR. Data represent at least two biological replicates per time point, with each PCR reaction run in triplicate. Expression levels of all transcripts were compared with housekeeping genes and the relative changes in gene expression were compared with those of untreated cells using the 2^−ΔΔCT^ method, and are expressed as fold change compared with no spirochete control. Error bars represent S.E.M.

**Figure 2:**
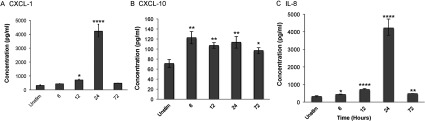
Protein expression in *B. burgdorferi* stimulated astrocytes Primary human astrocytes were stimulated with *B. burgdorferi* for 6, 12, 24, or 72 h. Supernatants from stimulated cells were collected, aliquoted and stored at −80°C. Levels of CXCL-1 (**A**), CXCL-10 (**B**), and IL-8 (**C**) were measured by individual ELISA according to the manufacturer's protocol (R&D Systems). Data represent two biological replicates per time point, with each sample run in triplicate. Error bars represent S.E.M.

**Figure 3:**
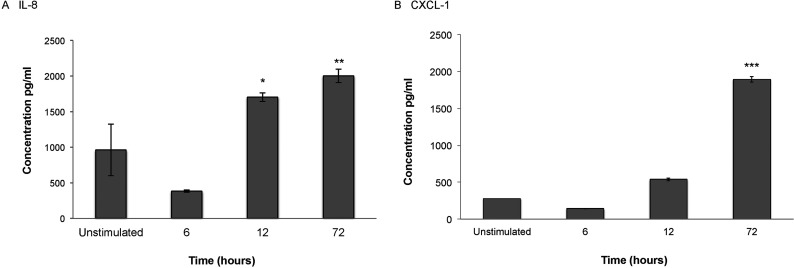
Protein expression in *B. burgdorferi* stimulated HBMEC Primary HBMEC were stimulated with *B. burgdorferi* for 6, 12 or 72 h. Supernatants from stimulated cells were collected, aliquoted and stored at −80°C. Levels of IL-8 (**A**), CXCL-1 (**B**) and CXCL-10 (not shown) were measured by individual ELISA according to the manufacturer's protocol (R&D Systems). Data represent two biological replicates per time point, with each sample run in triplicate. Error bars represent S.E.M.

To confirm whether *B. burgdorferi* induces chemokine production and secretion after co-incubation with primary HBMEC, we measured IL-8, CXCL-1, and CXCL-10 synthesis as above. IL-8 and CXCL-1 protein expression were maximal at 72 h (Figure 3). No CXCL-10 was detectable in unstimulated controls or in any treated samples until 72 h, where 65±29 pg/ml of the chemokine was detected in *B. burgdorferi* treated HBMEC.

## DISCUSSION

Chemokines and their receptors mediate trafficking of leukocytes into the CNS in response to injury or infection. A key feature associated with infection is disruption of the BBB, leading to enhanced neutrophil extravasation into the CNS (McColl et al., 2008). However, the contribution of brain microvasculature and glial inflammatory activity to the recruitment of systemic, potentially neurotoxic, inflammatory cells remains ill defined (Allen et al., 2012).

In this paper, we demonstrate that primary HBMEC and primary human astrocytes express a variety of chemokines and chemokine receptors in response to the spirochete, *B. burgdorferi*. These chemokines may contribute to recruitment of inflammatory cells and the onset and progression of neuronal damage leading to neuroborreliosis (Allen et al., 2012). Indeed, non-human primate astrocytes express the neutrophil chemoattractant IL-8 in response to *B. burgdorferi* (Ramesh et al., 2009). In regards to human patients, IL-8 is found in the CSF of individuals with neuroborreliosis (Grygorczuk et al. 2004; Henningsson et al., 2011). Although there are many potential sources of IL-8 in the CSF, we demonstrate that this key neutrophil chemoattractant is also made by human astrocytes in response to *B. burgdorferi*. In addition, we demonstrate that another neutrophil recruiting chemokine, CXCL-1, is also produced by astrocytes. Astrocytes possess the receptor for CXCL-1, allowing for an autocrine feedback loop that can prolong and amplify inflammation in the CNS (Dorf et al., 2000).

The precise role of astrocytes in the pathogenesis of Lyme borreliosis is unclear. In the macaque model of the disease, microglia are clearly involved in the early inflammation and elaborate a number of cytokines and chemokines; however, there is also significant evidence for astrocyte contribution to the inflammatory response to *B. burgdorferi*, both *in vivo* and *in vitro* (Ramesh et al. 2003; 2008; 2009). For example, primary rhesus macaque astrocytes stimulated with *B. burgdorferi* components elaborate both IL-6 and TNFα, which lead to astrocyte proliferation and apoptosis, respectively (Ramesh et al., 2003). Interestingly, we also saw up-regulation of TNFα from human astrocytes in response to *B. burgdorferi* at 6 h (Table 2); TNFα is a potent inducer of inflammatory responses and thus may serve to amplify the chemokine responses we observed. There is also evidence for astrogliosis in human neuroborreliosis, as evidenced by increased GFAP (glial fibrillary acidic protein) in the CSF of patients (Dotevall et al. 1996; 1999). These data suggest that in human patients, astrocyte proliferation and activation occurs in response to the spirochete, underlying the potential role of astrocytes in disease progression.

Likewise, HBMEC probably contribute to chemokine responses and subsequent inflammation in neuroborreliosis. Our results demonstrate that protein expression of both neutrophil chemoattractants CXCL-1 and IL-8 was strongly up-regulated by HBMEC in response to *B. burgdorferi*. Stimulation of endothelial cells from other anatomical sites with *B. burgdorferi* resulted in both robust IL-8 transcription (Burns et al., 1997), as well as CXCL-1 (Dame et al., 2007). *In vitro*, HBMEC stimulated with *Streptococcus pneumonia*e up-regulate IL-8 and CXCL-1, suggesting the elaboration of neutrophil-specific chemokines may be a common response to bacterial insults at the BBB (Banerjee et al., 2010). In previous studies, transfected HBMEC incubated with *B. burgdorferi* did not demonstrate up-regulation of cytokines or chemokines at 5 h post-stimulation by ELISA, suggesting that HBMEC do not store pre-formed chemokines and must synthesize these attractants *de novo* upon exposure to *B. burgdorferi* (Grab et al., 2007). The role of endothelial cells in neuroborreliosis has also been examined by observing spirochetal translocation across single-cell monolayers of transformed HBMEC (Grab et al. 2005; 2009). While these studies indicated a role for host plasminogen and Ca^2+^ signaling in translocation, there is scarce published data on the contribution of chemokines to the disruption of the BBB. However, the expression of inflammatory chemokines by the human BBB has not been well characterized *in vivo* or in multi-cell models *in vitro*, highlighting the need for further investigation (Holman et al., 2011).

The significance of increased expression of IL-8 and CXCL-1 by brain endothelia and astrocytes lies in their role in neutrophil recruitment into the CNS (Johnson et al., 2011). IL-8 has been shown to act as an endothelial chemoattractant for neutrophils and is directly involved with neutrophil migration on endothelial surfaces (Burns et al., 1997). Allen, et al., (2012) have demonstrated recently that migration of neutrophils through the neurovascular unit shifts the neutrophils to a neurotoxic phenotype resulting in increased release of proteases, inflammatory cytokines, chemokines, and NETs (neutrophil extracellular traps). They demonstrated that when neutrophils harvested immediately following CNS endothelial transmigration were co-cultured with primary cortical neurons, they induced significant neuronal loss within 30 min of application. Neutrophils that had not been activated by CNS endothelial transmigration demonstrate no neurotoxicity. Others have shown that co-culture of activated neutrophils with primary hippocampal neurons (Dinkel et al., 2004) or dissociated dorsal root ganglion neurons (Shaw et al., 2008) also resulted in massive neuronal loss. Thus, the *B. burgdorferi*-induced elaboration of IL-8 and CXCL-1 by astrocytes and HBMEC probably contribute to the neural damage associated with neuroborreliosis through recruitment of neurotoxic neutrophils.

CXCL-10, a chemokine important for T-cell accumulation, was also significantly up-regulated in *B. burgdorferi*-stimulated astrocytes and HBMEC. High levels of CXCL-10 are observed in the CNS of humans with neuroborreliosis (Henningsson et al., 2011). Indeed, it has been postulated that CXCL-10 creates a chemokine gradient between the CSF and serum and recruits CD4+ T-cells into the CSF of patients with neuroborreliosis (Lepej et al., 2005). CXCL-10 plays an important role in other CNS infections, and is one of the most highly expressed chemokines in response to West Nile Virus (Lim and Murphy, 2011). Interestingly, CXCL-10 and its receptor CXCR3 are expressed by neurons (Lim et al., 2011). We did not observe up-regulation of CXCR3 on astrocytes (results not shown); suggesting that, *in vivo*, neurons may be the primary cell type responding to this chemokine. Indeed, neuronal apoptosis is mediated by increased expression of CXCL-10 in response to a variety of infectious insults including simian HIV encephalitis (Sui et al., 2006). Clearly, the crosstalk between neurons and astrocytes in CNS infections is key to determining the ultimate outcome of the disease.

The fact that very few chemokines and related genes changed in response to stimulation with *B. burgdorferi* was unexpected. However, these data make sense in the context of a spirochetal pathogen. These organisms have a reputation for ‘stealth’ and persistence even in immunocompetent hosts. While possessing a number of pathogen associated molecular patterns and inflammatory stimulants like lipoproteins, *B. burgdorferi* lacks LPS (lipopolysaccharide) in its outer membranes. Therefore *B. burgdorferi’s* effects on glia and other supporting cells such as HBMEC, may be more subtle than that observed in acute, severe infections of the CNS.

Together these data and that presented herein show that *B. burgdorferi* can stimulate up-regulation of chemokines from brain microvascular endothelia and astrocytes, which potentiate entry of neurotoxic neutrophils into the brain. Such an event can lead to neuronal loss and may contribute to the cognitive impairments and other neurological deficits associated with neuroborreliosis. This is the first study to investigate comprehensively the chemokine-centered responses of primary human astrocytes and HBMEC to *B. burgdorferi*. Understanding the contributions of individual glia cell types to the damage induced by *B. burgdorferi* will ultimately allow for the development of targeted, cell type-specific interventions and therapies.

## Online data

Supplementary data
