# Supplementary material for: The multifaceted responses of primary human astrocytes and brain microvascular endothelial cells to the Lyme disease spirochete, Borrelia burgdorferi
Source: ASN Neuro. 2013 Aug 16;5(3):e00119. doi: 10.1042/AN20130010 (PMC3745032; doi:10.1042/AN20130010)
Supplement: Supplementary data [file an005e119add.pdf]

ASN NEURO RESEARCH ARTICLE

ASN NEURO 5(3):art:e00119.doi:10.1042/AN20130010

OPEN ACCESS

The multifaceted responses of primary human astrocytes and brain microvascular endothelial cells to the Lyme disease spirochete, *Borrelia burgdorferi*

Catherine A. Brissette*[1], Eric D. Kees*, Margaret M. Burke†, Robert A. Gaultney*, Angela M. Floden* and John A. Watt†
*Microbiology and Immunology, University of North Dakota School of Medicine and Health Sciences, Grand Forks, ND 58203, U.S.A.
†Department of Anatomy and Cell Biology, University of North Dakota School of Medicine and Health Sciences, Grand Forks, ND 58203, U.S.A.

SUPPLEMENTARY DATA

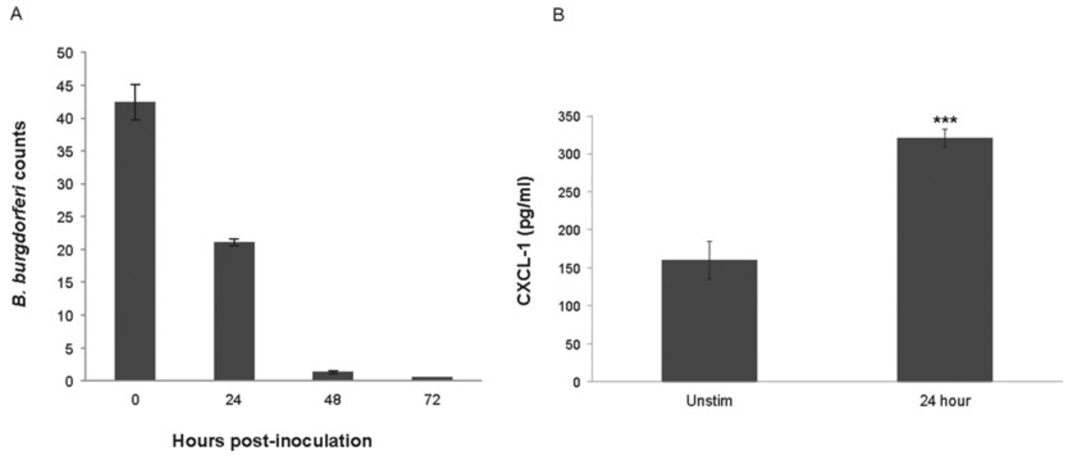

Figure S1 ***B. burgdorferi*** **viability during co-culture with astrocytes and response of astrocytes to dead bacteria**
(A) Primary human astrocytes were incubated with *B. burgdorferi* for 24, 48, or 72 h at 37°C in 5% CO_2. At each time point, aliquots of the culture medium were removed and examined by darkfield microscopy for motile spirochetes in ten random fields. Data represent triplicate culture samplings per time point. Error bars represent S.E.M. (B) Primary human astrocytes were incubated with heat killed *B. burgdorferi* for 24 h. Supernatants from stimulated cells were collected, aliquoted and stored at −80°C. Levels of CXCL-1 were measured by individual ELISA according to the manufacturer's protocol (R&D Systems). Data represent two biological replicates per time point, with each sample run in triplicate. Error bars represent S.E.M.

[1]To whom correspondence should be addressed (email catherine.brissette@med.und.edu).
© 2013 The Author(s) This is an Open Access article distributed under the terms of the Creative Commons Attribution Licence (CC-BY) (http://creativecommons.org/licenses/by/3.0/) which permits unrestricted use, distribution and reproduction in any medium, provided the original work is properly cited.
